# Genomic characterization of *Citrobacter freundii* clinical isolates from Tanzania

**DOI:** 10.1128/mra.00363-25

**Published:** 2025-07-02

**Authors:** Gerald Mboowa, Benson R. Kidenya, Ivan Sserwadda, Stephen Kanyerezi, Inyasi Lawrence Akaro, Baraka Mkinze, Jeremiah Seni

**Affiliations:** 1 Department of Immunology and Molecular Biology, College of Health Sciences, School of Biomedical Sciences, Makerere University 58588, Kampala, Uganda; 2 The African Center of Excellence in Bioinformatics and Data-Intensive Sciences, the Infectious Diseases Institute, College of Health Sciences, Makerere University, Kampala, Uganda; 3 Department of Biochemistry and Molecular Biology, Weill Bugando School of Medicine, Catholic University of Health and Allied Sciences 361475, Mwanza, Tanzania; 4 Department of Surgery, Weill Bugando School of Medicine, Catholic University of Health and Allied Sciences 361475, Mwanza, Tanzania; 5 Department of Microbiology and Immunology, Weill Bugando School of Medicine, Catholic University of Health and Allied Sciences 361475, Mwanza, Tanzania; Rochester Institute of Technology, Rochester, New York, USA

**Keywords:** *Citrobacter freundii*, whole-genome sequencing, antimicrobial resistance (AMR), virulence factors, genomic surveillance

## Abstract

We report draft genomes of three *Citrobacter freundii* isolates from Tanzania. Genomes averaged 6.9 Mb with 51.49% GC content and 114× depth. ST167 carried multiple extended-spectrum β-lactamase, fluoroquinolone, and aminoglycoside resistance genes alongside virulence factors, highlighting the need for genomic surveillance of emerging multidrug-resistant *C. freundii* lineages.

## ANNOUNCEMENT

*Citrobacter freundii* is a Gram-negative bacterium from the *Enterobacteriaceae* family, typically considered part of the human gut flora but increasingly implicated in healthcare-associated infections, particularly among immunocompromised patients (1). The emergence of multidrug-resistant strains and the acquisition of resistance genes via mobile genetic elements have heightened concern for infection control and antimicrobial stewardship programs.

As part of a genomic surveillance study, we investigated *C. freundii* isolates collected between January and May 2020 from orthopedic wards at Bugando Medical Centre, a 900-bed tertiary referral hospital in Mwanza, Tanzania. The isolates were part of a broader study aimed at evaluating the transmission of extended-spectrum β-lactamase (ESBL)-producing bacteria in clinical and hospital environments. A total of 283 participants were enrolled, including 265 index patients and 18 neighboring patients. Additional samples were collected from 18 non-medical caregivers, 24 healthcare workers, and 88 environmental surfaces (2).

Sample collection involved rectal or stool swabs from patients within 24 hours of admission. ESBL-producing Gram-negative bacteria were identified via selective culture on MacConkey agar supplemented with cefotaxime (2). Bacterial identification was done using standard biochemical tests, including oxidase, catalase, citrate utilization, and triple sugar iron agar reactions. Antimicrobial susceptibility testing was performed according to Clinical and Laboratory Standards Institute (CLSI) guidelines using the Kirby-Bauer disk diffusion method. The antibiotic panel included cefotaxime, ceftazidime, ciprofloxacin, gentamicin, meropenem, and trimethoprim-sulfamethoxazole. Confirmation of ESBL production was done using the combined disk method (2).

After presumptive ESBL-producing Gram-negative bacteria were identified on MacConkey agar with cefotaxime, the cultures were incubated aerobically at 35–37°C for 18–24 hours. Colonies with distinct morphology were subcultured for a purity check. Confirmed *Enterobacteriaceae* isolates underwent genomic DNA extraction using the QIAamp DNA Mini Kit (Qiagen, Germany) as per the manufacturer’s instructions. DNA quality and quantity were assessed using NanoDrop 2000 spectrophotometry (Thermo Fisher Scientific, USA). Subsequently, whole-genome sequencing was performed at the Earlham Institute (Norwich, UK) using the Low Input Transposase Enabled Illumina library preparation protocol on the NovaSeq 6000 platform, generating 150 bp paired-end reads.

Bioinformatic analysis was performed using the rMAP pipeline v1.0 (3) for species genomic confirmation, antimicrobial resistance (AMR) gene detection, MLST typing, and genome annotation. To validate and extend these analyses, the TheiaProk_Illumina_PE_PHB workflow v1.0.0 (4) on the Terra platform was used for quality control assessment, including QUAST v5.2.0 (5) and BUSCO v5.8.2 (6) evaluations, providing robust and reproducible genomic characterizations (Table 1). Quality control was performed with FastQC v0.11.9 (7) and Trimmomatic v0.39 (8). *De novo* genome assembly was performed using SPAdes v3.15.2 with default parameters optimized for Illumina paired-end data. SPAdes v4.2.0 (9) constructs the assembly using a de Bruijn graph approach and includes read error correction and scaffolding steps, making it well-suited for bacterial genomes. Genome annotation was performed using Prokka v1.14.6 (10), which enabled rapid functional annotation of assembled genomes, assigning gene functions and identifying coding sequences, rRNAs, and tRNAs.

**TABLE 1:** Genomic features of the three draft *C. freundii* genomes^*a*^^,^^*b*^

Strain	A55880	A56400	A56429
ST	ST116	ST167	ST263
Genome size (bp)	5,502,433	10,239,272	5,160,700
G + C (%)	51.61	51.18	51.67
Number of contigs	144	225	59
Size of the largest contig	503,328	273,666	836,533
N50 (bp)	173,136	13,036	276,703
Completeness			
Sequencing depth	133×	62×	147×
CDS (total)	5,228	9,484	4,825
Number of tRNAs	309	631	309
Drug resistance genes	*blaCTX-M-15, gyrA_T83I, blaCMY-181, blaTEM-1, aac (3)-IId, qnrB1, sul2, tet(A), tet(D), blaOXA-2, aac (3)-Ib, catB3, blaOXA-1, aac(6')-Ib-cr5, aadA2, dfrA12, sul1, dfrA14, mph(A), catA1, aadA1*	*blaCMY, qnrB38, gyrA_T83I, blaTEM-1, dfrA12, aadA2, sul1, mph(A), aac (3)-IId, tet(A), blaCTX-M-15, tet(B), catB3, blaOXA-1, aac(6')-Ib-cr5, catA1, emrD, blaEC, mdtM, acrF*	*qnrB, gyrA_T83I, blaCMY-97, blaTEM-1, blaCTX-M-15, tet(D), sul2, sul1, aadA16, dfrA27, arr-3, aac(6')-Ib-cr5, qnrB6*
Plasmid replicons	*IncFIB(pB171), IncFII(Yp), RepA*	*Col440I, IncFIA, IncFIB(AP001918), IncHI1B, IncR*	*RepA*
Virulence and stress genes	*pcoE, pcoS, pcoR, pcoD, pcoC, pcoB, pcoA, silP, silA, silB, silF, silC, silR, silS, silE, pcoS, fieF, arsR, arsD, arsA, arsB, arsC, merT, merA, merD, merE, qacF, merR, merT, merP, merC, qacEdelta1, merR*	*asr, fieF, fieF, ariR, terW, terZ, terD, arsC, arsC, arsR, qacEdelta1, arsA, arsD, arsC, arsB*	*fieF, qacEdelta1*
SRA accession number	SRR32995216	SRR32995217	SRR32995218

^*a*^ The three *C. freundii* genomes show distinct genomic features. Isolate ST263 (A56429) exhibits moderate antibiotic resistance and few virulence genes. ST167 (A56400) is highly resistant with diverse virulence factors and plasmids. ST116 (A55880) presents an intermediate profile with unique resistance mutations.

^*b*^ Abbreviation: CDS, coding sequences.

AMR genes were identified using AMRFinderPlus v3.12.8 (11) and ResFinder v 4.7.2 (12) with minimum identity and coverage thresholds set at 90% and 60%, respectively. Plasmid replicons were detected using PlasmidFinder v2.1.1 (13) with default parameters. MLST was determined via the PubMLST database using mlst v2.22.0(14).

**Fig 1:**
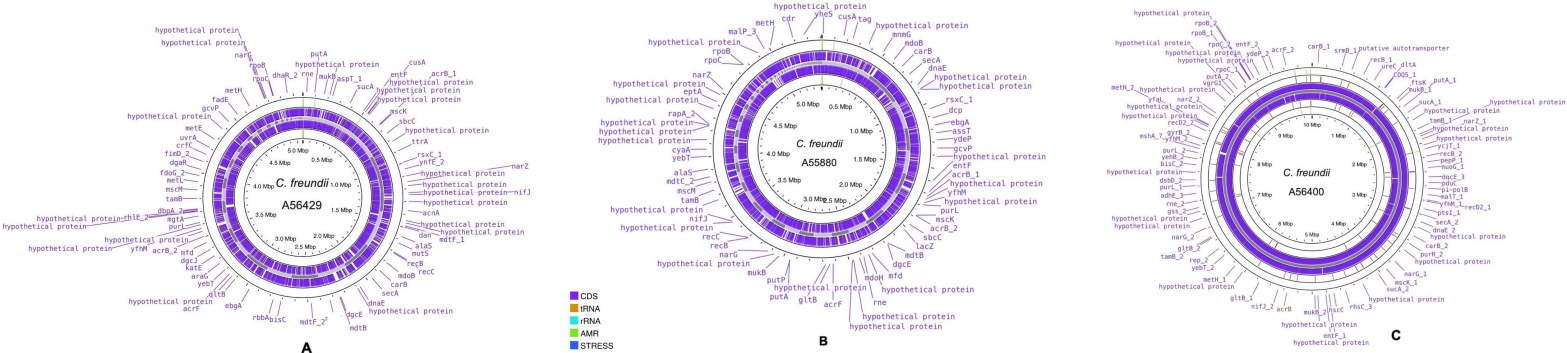
(A–C) Genome circularization of draft *C. freundii* A56429, *C. freundii* A55880, and *C. freundii* A56400. The concentric rings display annotated genomic features, with the outermost ring showing coding sequences (CDS) and genes, including hypothetical proteins, annotated by Prokka on both strands. Functional elements are color-coded: CDS (purple), tRNA (orange), rRNA (cyan), AMR genes (green), and stress response genes (blue). Visualization was performed using Proksee (https://proksee.ca/). Genomic coordinates are marked in megabase pairs (Mbp) around the circle.

*Three C. freundii* isolates (A55880, A56400, and A56429) were sequenced with an average depth of 114× and genome sizes ranging from 5.1 to 10.2 Mbp (Fig. 1A through C). The unusually large genome size (~10.2 Mb) of *C. freundii* A56400 may be attributed to the presence of multiple plasmids, integrated prophages, and horizontally acquired mobile genetic elements. GC content ~51.5%. MLST analysis identified the isolates as ST116, ST167, and ST263. ST167 (A56400) exhibited the broadest AMR profile, including *bla*CTX-M-15, *bla*TEM-1, *bla*CMY, *qnr*B, and *aac*(6′)-Ib-cr, alongside virulence genes *espX1*, *ybtQ*, and *ybtP* (Table 1). These findings indicate iron acquisition capacity and potential immune evasion via Type III secretion systems. ST263 and ST116 demonstrated moderate and intermediate profiles, respectively, with varying plasmid content. The most common plasmid replicons included IncFIB, IncHI1B, and IncR.

This study highlights the genomic diversity and resistance potential of *C. freundii* strains circulating in Tanzanian healthcare settings. The identification of ST167 as a high-risk clone, harboring diverse AMR and virulence determinants, underscores the need for continued genomic surveillance. These results provide critical insights into infection control strategies in resource-limited settings.

## Data Availability

The raw sequencing reads have been deposited in the Sequence Read Archive (SRA) under accession numbers SRR32995216, SRR32995217, and SRR32995218. All source code for the rMAP pipeline, installation instructions, and implementation can be accessed via GitHub (https://github.com/GunzIvan28/rMAP). The raw read files from this study are publicly available at the Sequence Read Archive (SRA) of the National Center for Biotechnology Information (NCBI) under the study BioProject ID: PRJNA1247136.
